# The effect of shock loading on the performance of a thermophilic anaerobic contact reactor at constant organic loading rate

**DOI:** 10.1186/2052-336X-12-84

**Published:** 2014-05-12

**Authors:** Elif Senturk, Mahir Ince, Guleda Onkal Engin

**Affiliations:** 1 Department of Environmental Engineering, Gebze Institute of Technology, Kocaeli, Turkey; 2 Department of Environmental Engineering, Yildiz Technical University, Davutpasa Campus, Istanbul, Turkey

**Keywords:** Thermophilic, Anaerobic contact reactor, Shock loading, Recovery, Stability

## Abstract

The influences of organic loading disturbances on the process performance of a thermophilic anaerobic contact reactor treating potato-processing wastewater were investigated. For this purpose, while the reactor was operated at steady state conditions with organic loading rate of 5.5 kg COD/m^3^ · day, an instant acetate concentration increase (1 g/L) was introduced to the reactor. During the shock loading test of acetate, it was observed that the overall process performance was adversely affected by all the shock loading, however, the system reached steady state conditions less than 24 hours of operation indicating that thermophilic anaerobic contact reactor is resistant to shock loading and be capable of returning its normal conditions within a short time period.

## Introduction

The wastewater from potato processing industry can be considered as a complex wastewater because of rather high concentrations of suspended solids, high content of insoluble COD fraction and significant quantities of potential foaming substances, such as proteins and fats [1,2]. Therefore these wastewaters could only be discharged into municipal sewer system or receiving media after the reduction of the pollutants to acceptable levels. These wastewaters are usually treated with various combinations of aerobic and anaerobic biological processes due to high concentrations of readily biodegradable compounds [1,3-6].

It is known that both the substrate retention time and the degree of contact between influent substrate and living microorganism population affect the performance of anaerobic reactors [6]. Both of these parameters are a function of the mixing conditions ensured in the reactor. Mixing provides a suitable medium for the microorganisms to remain in suspension, as well as, for the biogas produced to leave the system [7]. Additionally, mixing ensures heat transfer, and a homogeneous substrate distribution by preventing stratification and formation of surface crust [8].

Hydraulic retention time (HRT) is another important design parameter for digesters. For a given volume of wastewater, a shorter HRT is an indication of a smaller digester and, therefore, a more cost-effective solution. In order to reduce HRT, temperature or solid retention time (SRT) increase were applied previously [9]. High-rate processes come forward to overcome this drawback of anaerobic treatment [10,11].

The anaerobic contact reactor, a typical example of high-rate anaerobic processes, can be classified as the counterpart of the aerobic activated sludge process. Both reactors are characterised with a constant, mechanical mixing of substrate with recycled biomass. Anaerobic contact reactors have been used extensively in the food processing industry to treat typical high strength effluents with relatively high suspended solids [6,12]. These reactors can be operated under different temperature ranges. Temperature can affect biochemical reactions in a number of ways, i.e. reaction rates increase with increasing temperature by the Arrhenius equation [6]. Increased reaction rates would reduce retention times and therefore capital and operational costs would decrease. Moreover, increased organic solids destruction would decrease the waste sludge while yielding more biogas [6,13].

The aim of this study is therefore to examine the effect of high acetate concentration on the performance and stability of the thermophilic anaerobic contact reactor (TACR). For this purpose, the most important operational parameters such as pH, alkalinity, total volatile acid concentration and biogas composition were monitored.

## Materials and methods

### Wastewater source and characterisation

The wastewater, used in this study, was obtained from a potato-processing factory. The wastewater was taken from the pipeline just after peeling and cutting processes were carried out. The characteristics of the wastewater can be found in Table 1. The COD/N/P ratio of the wastewater used was found to be 275/10/1.

**Table 1:** The characteristics of the wastewater used (after peeling and cutting processes)

**Parameter**	**Unit**	**Average**
TCOD	g/L	5.50
BOD_5_	g/L	4.50
Alkalinity	g CaCO_3_/L	2.25
pH	-	7.50
Total Kjeldahl nitrogen	g/L	0.23
Sulphate	g/L	0.45
Total solids	g/L	4.90
Total volatile solid matter	g/L	4.45

### Thermophilic anaerobic contact reactor configuration and operation conditions

**Figure 1:**
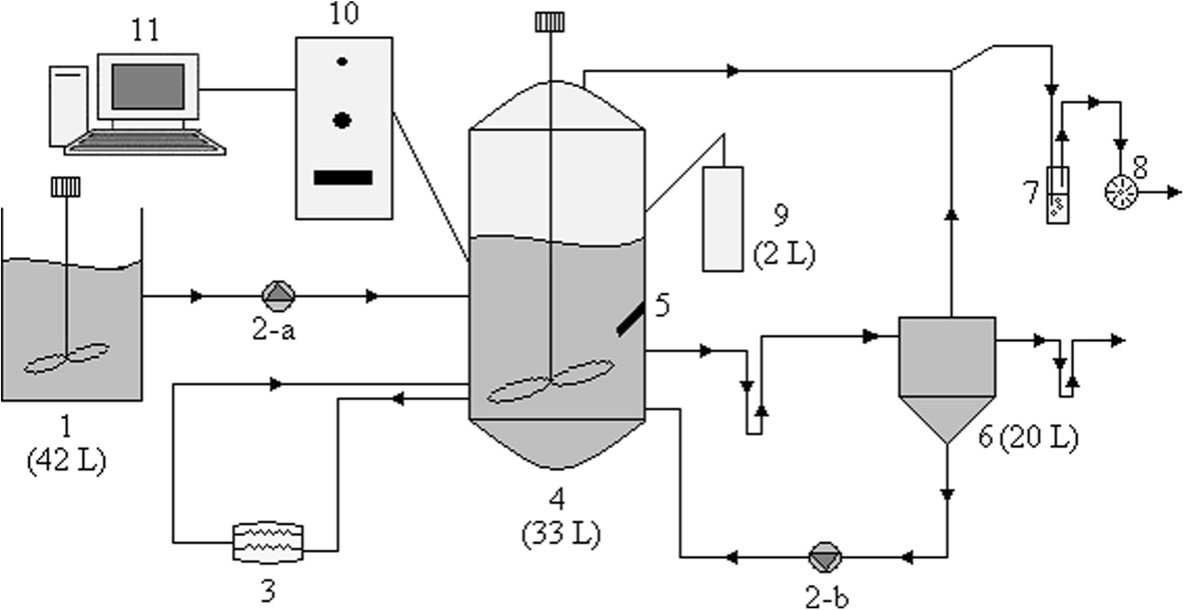
The schematic view and the flow chart of the TACR used in this study; 1) Feed tank, 2- Peristaltic Pump, 3) Heater, 4) Thermophilic Anaerobic Contact Reactor - TACR, 5) pH-meter, 6) Separation Tank, 7) Gas washing, 8) Gas-meter, 9) NaOH tank, 10) PLC Panel, 11) Computer.

The thermophilic anaerobic contact reactor (TACR) used in this study can be seen in Figure 1. The contact reactor and all the other components of the system were made of stainless steel. The contact reactor was constructed as a completely closed jacketed vessel, so that it was leak-proof and resistant to pressures up to 2 bars. The piping was installed using teflon and stainless steel pipes that are resistant to pressure and acidic/basic conditions. The control volume of the contact reactor was 33 L. Working with such a reactor would allow possible scaling up from laboratory scale to full scale easily. The feed tank was mixed continuously at a rate of 80 rpm to avoid precipitation of the particulate matter (starch). Anaerobic granular sludge taken from an anaerobic digester of a sewage treatment plant in a potato-processing factory was used as inoculum. In order to keep the reactor at thermophilic conditions, i.e. 55 ± 2°C, a 10 L heater tank was attached to the system. For this purpose, three PT100 temperature sensors were used. The pH of the system was monitored and controlled continuously with a pH probe and the pH value was adjusted by NaOH when necessary. The water used in gas washing was acidified to pH 3 by the addition of HCl and NaCl in order to prevent biogas dissolution. In order to prevent microorganism loss, a heat-insulated precipitation tank was also attached to the system. For the overall control of the system, a programmable logic controller (PLC/Siemens S7 300) was used, and data acquisition and visualization was carried out using WinCC SCADA (Siemens).

In order to study the effects of different operational parameters, the TACR was continuously operated for over a year (Şentürk et al., 2010), before the organic shock loading study was carried out. In order to examine the effect of high acetate concentration on the performance and stability of the TACR, acetate concentration in the reactor was increased to 1 g/L instantly, when the reactor was operated at organic loading rate of 5.5 kg COD/m^3^ · day (HRT = 1 day). During the feeding of shock acetate loading to the system, no other changes were made in the raw wastewater characteristics or flow rate. The response of the anaerobic culture in the reactor to this high acetate concentration was then observed at constant organic loading rate.

### Analytical methods

All the chemicals used were of analytical reagent grade and water used during the experiments was laboratory distilled water. The analytical methods, which were used in order to monitor the performance of the system, were performed using the methods given in the Standard Methods [14]. The COD and BOD_5_ analyses were carried out according to the STM 5220 C and STM 5210 B methods, respectively [14]. The TKN analyses were performed using the STM 4500-Norg B Macro-Kjeldahl [14]. The sulphate analyses were carried out using the STM 4500-SO_4_^2-^ method [14]. The alkalinity and total volatile fatty acid concentrations were determined according to STM 2320 B and STM 5560 C methods, respectively [14]. Acetic acid concentrations were conducted by a Gas Chromatography (Agilent) equipped with FID detector and a Zebran ZB-Wax capillary column, 30 m × 250 μm × 0,50 μm. Helium was used as the carrier gas. The oven temperature was initially set at 100°C for 1 min increasing 20°C/min to 120°C and then increasing 6.13°C/min to 205°C. The total duration was 15.87 minutes. The detector temperature was 240°C. The samples taken from the reactor were centrifuged for 15 minutes at 10000 rpm at room temperature and the supernatant of the sample was analysed accordingly. Additionally, the total solid matter and total volatile solid matter concentrations were also determined (STM 2540 B and STM 2540 C methods) [14].

The biogas produced was measured cumulatively using a gas-meter (Ritter) and the components (CH_4_, CO_2_, H_2_) were analysed by a Gas Chromatography (Agilent) using HP Plot Q + Molecular Sieve column, 60 m × 530 μm × 400 μm. Argon was used as the carrier gas with a gas flow of 4 mL/min. The oven temperature was initially set at 50°C for 5 min increasing 5°C/min to 80°C and kept at 80°C for 3 minutes, then increasing 10°C/min to 100°C. The total duration was 16 minutes. The temperature of TCD (Thermal Conductivity Detector) was 200°C.

## Results and discussions

This study investigated the adverse effects of high acetate loading on a high-rate anaerobic contact reactor operated under thermophilic conditions. The organic shock loading was introduced to the system by dramatic increase of acetate concentration. It should be noted that before the organic shock loading study, the reactor was continuously run under steady-state conditions for over a year. The findings are discussed in the following sections.

### pH and alkalinity

During the operation of a digester, pH is one of the most important factors and it is well known that anaerobic microbial activity is the highest in the pH range of 6.8 – 8.5 [15-17]. Additionally, the alkalinity of the anaerobic reactor should be maintained as close to the operating range as possible and there might be a need for addition of alkaline solutions in order to adjust pH especially during the acetogenesis phase. The pH fluctuations can affect both the bacterial growth and their activity in organic matter degradation adversely [18]. Therefore, to keep pH in a specified region is of importance.

**Figure 2:**
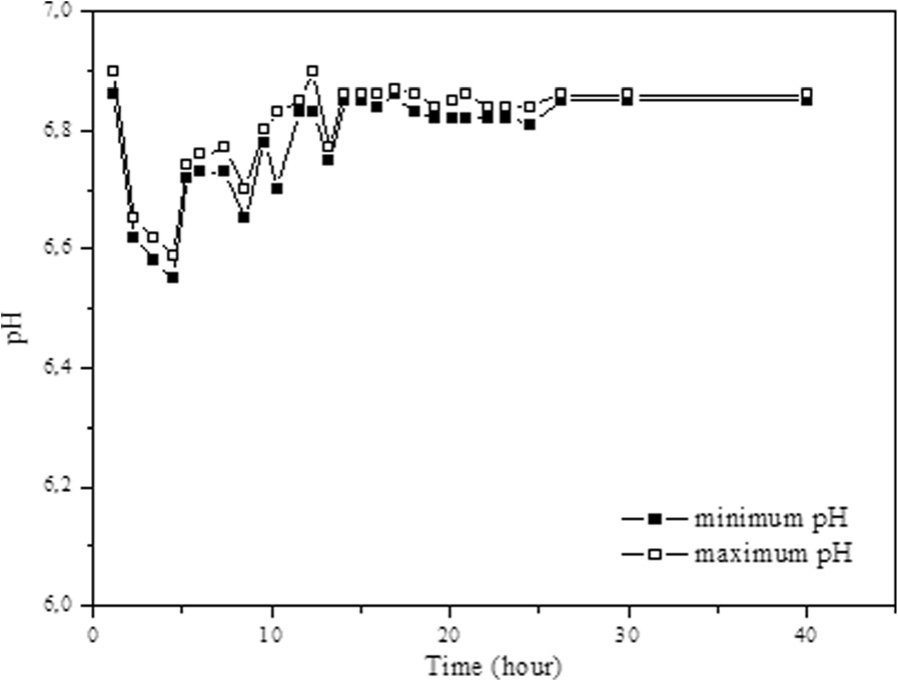
pH fluctuation after acetate addition.

The system's response to acetate loading was monitored from the point of pH variations. As can be seen from Figure 2, the pH fluctuation after the addition of high concentration acetate was not significant due to buffering capability of wastewater used. The decrease in pH in the initial period after the shock loading can be associated to an increase in free hydrogen and acetate concentration in the reactor which is a result of the imbalance between fermentation bacteria and methanogens [19-21]. After the first 5 hours, the pH of the system began to increase to reach its steady-state values. 14 hours after instant high concentration acetate addition, the fluctuation in pH ceased and the system reached steady state conditions. Less extensive oscillations were observed in the pH values, due to the settlement tank used in the system. Throughout the study, alkalinity fluctuations rarely encountered, indicating buffering capability of the system. During the study, the VFA/Alkalinity ratio (0,10 – 0,30) was almost always below the limit value of 0,33 [22].

### Volatile fatty acids

**Figure 3:**
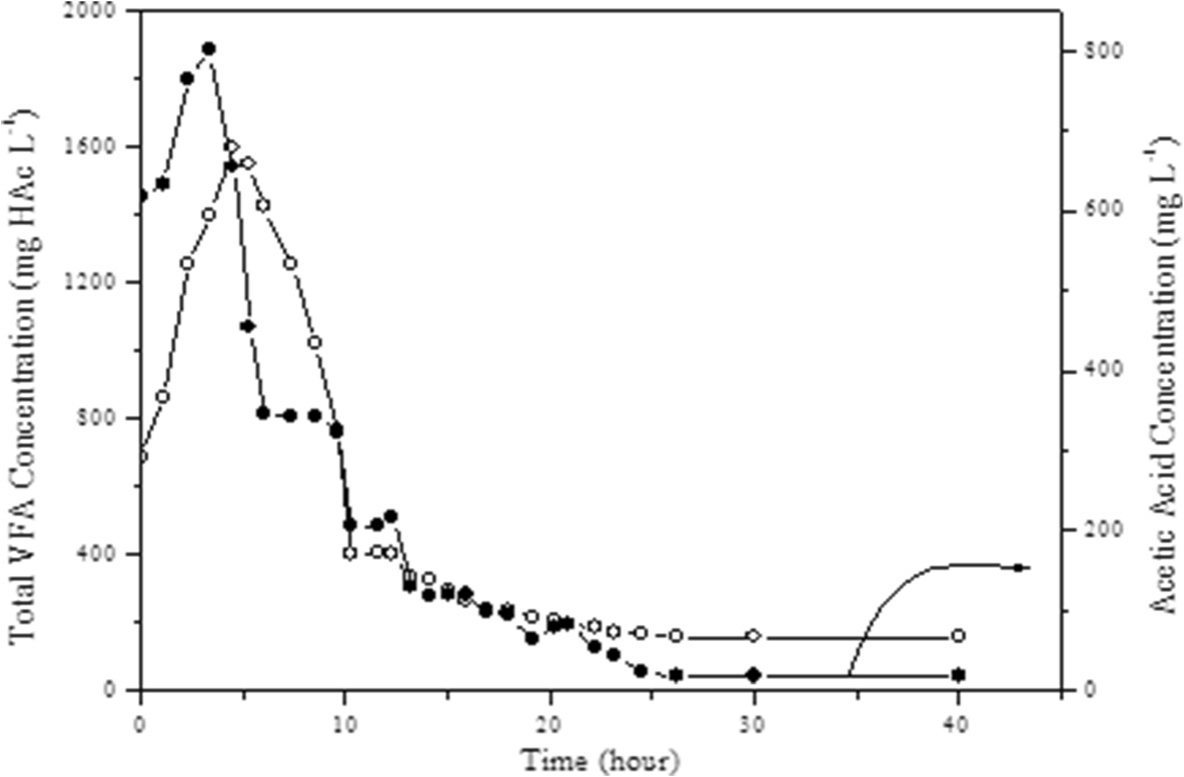
Effluent total VFA and acetic acid concentration.

In this study, another important parameter which needs to be observed was VFA. The total VFA and effluent acetic acid concentration change with time is given in Figure 3. As can be seen from Figure 3, the high values of both total VFA and acetic acid concentrations began to decrease after 5 hours of operation due to rapid conversion of acetate into methane by methanogens. The shock loading effect was tolerated due the use of contact reactor with a settlement tank. Another important result of this study was that the increase in acetate concentration and the total VFA concentration at the initial hours had quite similar trends indicating other VFA components in the system was insignificant. The GC analyses of volatile fatty acids confirmed this, as well. The total VFA concentration reached the steady state conditions within 24 hours.

### Biogas

Biogas composition varies depending on feedstock, digester type and chemical addition [23]. Additionally, the amount of biogas produced during anaerobic digestion depends on the feed organic matter content, the total volatile solid matter and the C/N ratio. The most known composition of biogas has about 60% methane, 35% carbon dioxide and about 5% the other gases [24,25].

**Figure 4:**
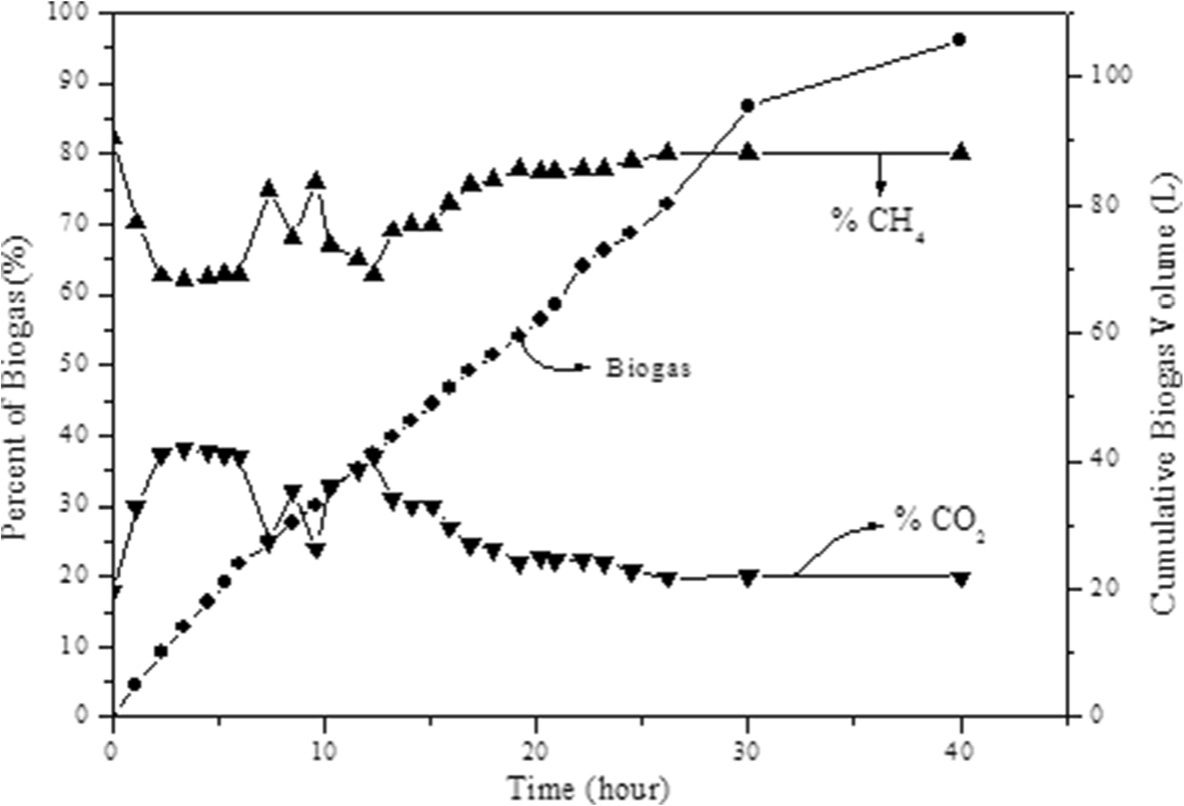
Cumulative biogas and percent of composition of biogas.

The cumulative biogas amount generated in the reactor and major constituents of biogas composition are given in Figure 4. Because HRT was low (0,96 day), fermentative hydrogen producing bacteria might have washed out from the reactor [26]. However, it was reported that hydrogen gas is produced via oxidation during the conversion of long-chain fatty acids [12]. In the case of our study, it was thought that hydrogen-consuming microorganisms, such as methanogens reduce the hydrogen gas produced during this conversion. The deterioration of hydrogen gas production with increasing substrate concentration was well in accordance with other studies [27-29]. As can be seen from Figure 4, after loading, biogas generation increased. Increase in cumulative biogas amount was the highest within the first few hours after acetate loading, indicating that methanogens were able to use acetate rapidly. It was observed that biogas generation decreased in parallel to acetate usage. After 20 hours of operation, the steady state conditions were reached. With regard to the composition of produced biogas, it was significant that, within the first two hours, although methane percentage in biogas decreased, carbon dioxide percentage increased due to generation of carbon dioxide as a result of used alkalinity by volatile fatty acids. After the first 2 hours, gas composition was observed to reach stable values and no change in gas composition was observed for about 5 hours.

However, fluctuations in biogas composition started again after 7 hours of operation and these fluctuations continued for about 20 hours. Only after 14 hours, methane percentage in biogas started to increase. However, the negative impact of high acetate concentration on methane bacteria did not last long and steady state conditions were reached after the first 20 hours of operation following shock loading.

## Conclusions

One of the most important features of the anaerobic contact reactors is that they are completely mixed reactors having a settlement tank. Anaerobic reactors, with continuous mixing facilities, are considered to be high-rate reactors from the point of mass transfer between substrate and microorganisms. Owing to this characteristic, the anaerobic contact reactor used in this study was found to be less affected by organic shock loading. When overall data were evaluated, thermophilic anaerobic contact reactor was found to be resistant to shock loading and can become stable only about in 20 hours.

## Competing interests

The authors declare that they have no competing interests.

## Authors’ contributions

ES and MI carried out all experimental design, experimental work, data analysis and data interpretation. GOE extended help in data interpretation and supervised the study. All authors have read and approved the final manuscript.
